# Impact of the policy environment on substance use among sexual minority women

**DOI:** 10.1016/j.dadr.2022.100058

**Published:** 2022-04-29

**Authors:** Laurie A. Drabble, Cat Munroe, Amy A. Mericle, Sarah Zollweg, Karen F. Trocki, Katherine J. Karriker-Jaffe

**Affiliations:** a San Jose State University College of Health and Human Sciences, San Jose, CA, USA; b Alcohol Research Group, Public Health Institute, Emeryville, CA, USA; c Columbia University School of Nursing; d Community Health & Implementation Research Program, RTI International, Berkeley, CA, USA

**Keywords:** Alcohol, Marijuana, Tobacco, Sexual minority women, LGBTQ, State policy, Recreational marijuana laws

## Abstract

**Background:**

Sexual minority women (SMW) are at greater risk for heavy episodic drinking, frequent marijuana use, and tobacco use than heterosexual women. Because past research has suggested the political and social environment may influence disparities in substance use by sexual orientation, this study examined associations of the U.S. state-level policy environment on substance use by SMW.

**Methods:**

A total of 732 SMW participants were recruited from two national online panels: a general population panel (*n* = 333) and a sexual minority-specific panel (*n* = 399). Past year substance use was defined by number of days of heavy episodic drinking (HED; 4+ drinks in a day), weekly tobacco use (once a week or more vs. less or none), and weekly marijuana use (once a week or more vs. less or none). Comprehensive state policy protection was defined by enactment of five policies protecting rights of sexual minorities. Regression models compared substance use outcomes for SMW living in states with comprehensive policy protections to SMW living in states with fewer or no protections. Models also assessed the impact of state policies related to alcohol (state monopoly on alcohol wholesale or retail sales), tobacco (state enactment of comprehensive smoke-free workplace laws) and marijuana (legalization of purchase, possession, or consumption of marijuana for recreational use).

**Results:**

Comprehensive policy protections were associated with fewer HED days. Recreational marijuana legalization was associated with higher odds of weekly use.

**Conclusions:**

Findings underscore the importance of policy protections for sexual minorities in reducing substance use, particularly HED, among SMW.

## 1. Introduction

Heavy episodic drinking (HED), drug use, and tobacco use are greater among sexual minority populations (e.g., lesbian/gay, bisexual, queer) relative to heterosexuals, and disparities in prevalence of substance use by sexual orientation are most consistent and pronounced among women (Hughes et al., 2020; Schuler et al., 2020). Sexual minority women (SMW) are at greater risk than heterosexual women for “binge” or heavy episodic drinking (Hughes et al., 2020), which is defined for women as drinking four or more drinks on a single occasion. Among SMW, HED is associated with negative health and social harms including alcohol dependence, injury, job loss, and heart attack or stroke (Dawson et al., 2005, 2012).

Relative to heterosexual women, SMW also have higher rates of tobacco use (Lee et al., 2009; McCabe et al., 2018) and marijuana use (Hughes et al., 2020), behaviors that are also associated with negative health outcomes, such as dependence and respiratory problems (Campeny et al., 2020; Omare et al., 2021). In the U.S., daily smoking is declining and light smoking (smoking 1–39 cigarettes, or less than two packs, per week) and low-rate smoking (fewer than 5 cigarettes a day) are increasing; however, daily and light smoking both appear be associated with parallel adverse health outcomes, particularly for cardiovascular disease (Schane et al., 2010). Research on marijuana use is mixed, with some studies documenting associations of marijuana use with physical problems and mental health, and others suggesting beneficial effects or null findings; for example, weekly marijuana use may be associated with lower self-rated mental health, but not lower self-rated physical health or quality of life (Lee et al., 2020). Although marijuana legalization has been important for addressing criminal justice and social inequities, understanding the possible impact of legalization on increased use is important for developing appropriate strategies for reducing potential unintended harm (Cerdá et al., 2020).

Structural stigma (defined as policies and norms at the societal, institutional and cultural level that negatively affect the opportunities, access, and well-being of a particular group) has emerged as an important factor in explaining disparities in risk for substance use by sexual orientation (Hatzenbuehler, 2014, 2016). For example, research in the U.S. found that political and social support for same-sex marriage was associated with lower rates of HED and tobacco use among sexual minorities (Everett et al., 2016; Hatzenbuehler et al., 2017, 2010). Similarly, living in states with comprehensive policies protecting the rights of sexual minorities (e.g., inclusion of sexual minorities as a protected category in hate crime laws and prohibiting discrimination based on sexual orientation in employment, housing, and public accommodations) was found to be associated with reduced risk for HED (Drabble et al., 2021) and reduced tobacco use (Titus et al., 2021) for sexual minorities relative to counterparts living in states with no or weak protections. Further, residence in states with policies that permit discrimination based on sexual orientation were associated with adverse health outcomes among sexual minorities. For example, prior to the 2015 extension of marriage rights to all same-sex couples in the U.S., research found greater evidence of psychological distress and worse self-reported health among sexual minorities living in states that prohibited same-sex marriage compared to those living in states that legalized it (Carpenter et al., 2018; Frost and Fingerhut, 2016; Gonzales and Ehrenfeld, 2018; Hatzenbuehler et al., 2010; Kail et al., 2015; Kennedy and Dalla, 2020; Raifman et al., 2017; Riggle et al., 2009). Policy protections for sexual minorities appear to positively impact health outcomes among sexual minorities, but have no impact – or only modest positive impact – on heterosexuals (Drabble et al., 2021; Hatzenbuehler et al., 2010; Solazzo et al., 2018; Titus et al., 2021). Living in states that enacted policies allowing denial of services to same-sex couples for religious reasons (e.g., permitting adoption agencies to deny same-sex couples or allowing government officials to refuse marriage licenses to same-sex couples) was associated with greater psychological distress among sexual minorities, but had no impact on heterosexuals (Raifman et al., 2018). The possible effects of policy protections on marijuana use has been under-investigated and is inconclusive (Drabble et al., 2021).

Research focusing on the impact of policy environments for SMW is important for several reasons. First, although research has documented disparities in substance use by sexual orientation across sex and gender identities, disparities are greater and more persistent among SMW compared to heterosexual women than differences by sexual orientation among men (Blosnich et al., 2013; Hughes et al., 2020; Johnson et al., 2016; Lee et al., 2009). These disparities underscore the importance of investigating both individual and environmental factors associated with substance use among SMW to inform intervention efforts. Second, policy contexts may be particularly salient for substance use among SMW, and this important topic warrants further investigation. For example, one study found living in states with nondiscrimination laws was associated with reduced disparities in HED among SMW relative to heterosexual women, but no differences were found by sexual orientation among men (Greene et al., 2020). Another study found strong alcohol policy environments were significantly associated with reduced HED among SMW, but not among men (Greene et al., 2021a). Third, there is a need to explore the impact of policies on substance use among SMW that, to date, have been under investigated. Philbin et al. (2019) examined marijuana use and marijuana use disorder by sexual orientation and residence in states with and without medical marijuana laws (MML) and found fewer differences by MML status among sexual minority men than among SMW. They noted a need for research on possible relationships between recreational marijuana laws and marijuana use among sexual minorities.

Finally, there is a need for studies that address methodological gaps in research to date. Few studies have concurrently evaluated the impact of state-level policies designed to protect the rights of sexual minorities and state-level policies that may impact substance use, such as policies for regulating alcohol and marijuana sales or creating smoke-free environments. One notable exception was a study by Greene and colleagues (Greene et al., 2021b), which explored the protective effect of both state-level nondiscrimination policies and the alcohol policy environment on HED among sexual minority adults in the U.S. They found no significant associations among men, but HED was lower among women who lived in states with both stronger alcohol policies and inclusive nondiscrimination laws. In states without inclusive nondiscrimination policies, odds of HED were greater among sexual minority than heterosexual women, and the alcohol policy environment did not influence that relationship. These studies assessed any HED in the past 30 days, with the authors calling for future research using additional and stronger measures of heavier alcohol use in relation to both the alcohol policy environment and the presence of inclusive policy protections for sexual minorities.

Studies on policy and substance abuse outcomes that disaggregate bisexual and lesbian women are also needed. Many studies suggest HED, tobacco use, and marijuana use may be higher among bisexual women than lesbian women (Evans-Polce et al., 2020; Hughes et al., 2020; McCabe et al., 2021, 2004, 2018; Philbin et al., 2019; Schuler and Collins, 2020; Shokoohi et al., 2021). A majority of studies examining the impact of structural stigma and state policy on sexual minority populations combine sexual orientation categories in analyses (Hatzenbuehler et al., 2009, 2010; Raifman et al., 2018; Woodford et al., 2015), but some studies suggest the impact of policies on health may be greater for bisexual than lesbian women (Philbin et al., 2019). Others have found no significant differences in health impacts of state policy between lesbian and bisexual women (Everett et al., 2016).

The current study addresses gaps in the extant literature by examining how past year number of HED days, tobacco use, and marijuana use among SMW may be associated with the concurrent presence of comprehensive policy protections for sexual minorities and by the presence of state-level policies related to regulation of alcohol sales, tobacco use, and recreational marijuana use in the United States (U.S.).

## 2. Methods

### 2.1. Data and sample characteristics

Data for this study were drawn from a larger project focusing both on methodological approaches for sampling and factors impacting substance abuse among SMW. SMW participants in the current study were recruited from two national online panels: a general population panel (*n* = 333) and an LGBT-specific panel (*n* = 399). Eligibility for participation in the panel samples was restricted to participants over the age of 18 who identified as lesbian, bisexual, or queer; resided in the U.S.; and described themselves as female. The general panel had only a binary male/female option for demographic data and did not assess whether respondents were assigned female at birth. Although the LGBT-specific panel allowed participants to select multiple sex and gender identities, eligibility was restricted to individuals who selected “female” as at least one of the response options in order to ensure comparability with the general panel sample. We refer to participants as “women” in this paper, although we acknowledge that study participants may have endorsed other categories had they been provided such options.

Recruitment was designed to oversample SMW who identified as African American/Black or Latinx. Oversampling these groups ensured we would have an adequate sample size to detect potential differences by race/ethnicity to achieve additional research aims of the project not described here. Specifically, recruitment targeted a random stratified sample that was 1/3 African American/Black, 1/3 Latinx, and 1/3 unspecified race and ethnicity (any race/ethnicity). Data were collected in four waves (using the stratified approach described above) over the summer and fall of 2019. Table 1 provides an overview of sample demographics.

### 2.2. Measures

#### 2.2.1. State policy environment

##### 2.2.1.1. Comprehensive protections for sexual minorities.

To create an indicator of comprehensive protections, we first created an index comprised of eight policies relevant to sexual minorities, which were adapted from the Movement Advancement Project (Movement Advancement Project, 2015). The index included five policies designed to protect the rights of sexual minorities including the following: 1) prohibition against discrimination based on sexual orientation by employers (both private and public/government); 2) housing non-discrimination laws inclusive of sexual orientation; 3) laws prohibiting discrimination based on sexual orientation in public accomodations (including protection against unfair refusual of service in, denial of entry to, or other explicit discrimination in places accessible to the public, such as stores, restaurants, parks, hotels, medical offices, and banks); 4) hate crime laws that explicitly include sexual minorities; and 5) laws prohibiting discrimination in adoption, foster parenting, or both, based on sexual orientation of parent(s). The index also included three potential negative policies: 1) religious exemption laws permiting discrimination in services (e.g., health care, private businesses, state officials who decline to marry same-sex couples) based on religious or moral grounds; 2) policies that allow denial of adoption and/or foster parenting by same-sex couples; and 3) state bans on cities/counties passing nondiscrimination protections based on sexual orientation. Laws that prohibit cities and counties from extending local nondiscrimination laws to classes not already included in state law have been used to prevent cities from passing policies protecting sexual or gender minority people from discrimination, or to nullify local ordinances designed to extend protections to sexual or gender minorities. Each positive policy was assigned a value of 1 and each negative policy was assigned a value of −1. Items on the index were summed, resulting in a possible score ranging from −3 to 5.

We constructed a dichotomous variable to compare respondents living in states (*n* = 15 states and the District of Columbia) with comprehensive policy protections (score of 5, with no negative policies) to those living in states with limited or no protections and/or those with one or more negative policies (score of less than 5). Our rationale for the dichotomous construction was threefold. First, the primary aim of the study was to examine the potential protective effects of supportive policies on behaviors. Second, we sought to extend the work of prior studies that used dichotomous measures to examine health impact on sexual minorities of “comprehensive policy protections,” “high policy support” or presence of state policies prohibiting discrimination based on sexual orientation (Drabble et al., 2021; Gonzales and Ehrenfeld, 2018; Greene et al., 2021a; Hatzenbuehler et al., 2020; Solazzo et al., 2018). The construction of our dichotomous comprehensive policy protection variable also aligns with the designation of “high equality states” in the Movement Advancement Project’s classification scheme. Third, we included negative policies in the construction of the index and dichotomous variable because of prior research documenting the harmful impact of even one negative policy, such as religious exemption laws (Raifman et al., 2018) or bans against same-sex marriage (Hatzenbuehler et al., 2010; Kail et al., 2015).

##### 2.2.1.2. Substance use policies.

We also created dichotomous indicators for the state policy environment in 2019 specific to each substance. Alcohol policy environment was constructed as a dichotomous variable based on data from the Alcohol Policy Information System (APIS), which provides detailed information about a variety of alcohol-related policies in the U.S. Use of a binary variable of state monopoly on retail or wholesale alcohol sales (yes/no) has been validated as a robust predictor of alcohol related harms, and was the strongest predictor among several alcohol policy variables (which also included state level taxes on spirits or beer, state level policy allowing off-premise alcohol retail sales after 10 p.m., and local density of liquor stores and bars) (Trangenstein et al., 2020). We constructed a variable indicating whether states used a state-run wholesale or retail distribution system for at least one alcohol beverage subtype (spirits, wine, beer; *n* = 17 out of 51 states, including Washington, DC).

Legalization of purchase, possession, or consumption of marijuana for recreational use was also constructed as a binary variable based on data from APIS (*n* = 11 out of 51 states). We focused explicitly on legalization of recreational marijuana because this topic has been under-investigated in relation to SMW (Philbin et al., 2019). Furthermore, legalization of recreational marijuana is associated with increased marijuana use relative to both states without legalization and states with only medical marijuana legalization, but there is a dearth of research on impacts among potentially vulnerable subpopulations (Cerdá et al., 2020).

Tobacco policy was drawn from data from the American Lung Association. We created a binary variable based on whether states had comprehensive smoke-free workplace laws (yes/no), which prohibit smoking in all public places and workplaces, including restaurants and bars (*n* = 28 out of 51 states). We selected this variable based on research documenting a robust protective relationship of comprehensive smoke-free laws with tobacco use including reduced initiation, lower prevalence of smoking, as well as reduction in amount of smoking (Apollonio et al., 2021; Azagba et al., 2020). Comprehensive smoke-free laws also demonstrated stronger effects than tobacco taxes across a wider range of smoking patterns (Apollonio et al., 2021).

#### 2.2.2. Substance use measures

##### 2.2.2.1. Number of 4-plus drinking days.

We used a graduated frequency (GF) measure that assessed frequency of drinking in a graduated series of quantity intervals (Greenfield, 2000). The series of questions began with a definition of a “drink” for participants: “Think of all kinds of alcoholic beverages combined, that is, any combination of bottles or cans of beer or malt beverages, glasses of wine, or drinks containing liquor of any kind.” In this question, 1 drink is equal to a 12- ounce bottle or can of beer, a 5-ounce glass of wine, or 1 shot of liquor (1.5 ounces). The survey then asked about the number of drinking days in the past year using the following quantity intervals: consumption of 12 or more drinks in one day; at least 8 but less than 12 drinks; 5, 6, or 7 but no more than 7 drinks; 4 drinks; 3 drinks; 2 drinks; and 1 drink. For example: “During the last 12 months, how often did you have 4 drinks but no more than 4 drinks of any kind of alcoholic beverage (in a single day), that is, any combination of bottles or cans of beer, glasses of wine, or drinks containing liquor of any kind?” Frequency was measured using a 7-point scale: Every day or nearly every day; 3–4 times a week; once or twice a week; 1–3 times a month; less than once a month; once in those 12 months; never in those 12 months. Consistent with the GF approach (see Greenfield et al. 2000), we constructed a continuous variable of the total number of days in the past year that participants consumed 4 or more drinks in the same day. Specifically, for each daily quantity of alcohol endorsed (e.g., 12 + drinks; 8–11 drinks), the reported frequency is standardized to reflect the corresponding number of drinking days in a year, set at the midpoint of the range. For example, a participant who reported consuming a given volume of alcohol “3 to 4 times a week” would be coded as engaging in that behavior 180 days/year. Working highest to lowest, a series of sums were created reflecting the total number of days drinking a given volume of alcohol (or more) in a year. The summation is “capped” if the number of drinking days reaches 365. Using 4-plus drinking days as a measure of HED is consistent with the definition of exceeding daily drinking limits (no more than 3 drinks for women) issued by the National Institute on Alcohol Abuse and Alcoholism (Dawson et al., 2005).

##### 2.2.2.2. Weekly marijuana use.

Participants were asked, “How often have you used marijuana, hash, pot, THC or ‘weed’ during the last 12 months?” with the following response options: every day or nearly every day; about once a week; once every 2 or 3 weeks; once every month or two; less often than that; and never. Past 12-month weekly marijuana use was dichotomized as use once a week or more often vs. less frequent or no use.

##### 2.2.2.3. Weekly tobacco use.

Past 12-month tobacco use constructed based on a question about how often participants smoked tobacco cigarettes or used any other kinds of tobacco in the past 12 months with the following response options: daily or nearly every day, 1 to 4 days per week, once every 2 to 3 weeks, once every month or so, less often than that, and never. Tobacco use was dichotomized as use once a week or more often vs. less frequent or no use. Because light smoking is increasing in the U.S., and it is associated with adverse health outcomes that parallel those of daily smokers (Schane et al., 2010), this construction was designed to include weekly smokers as well as daily tobacco users in the focal category.

#### 2.2.3. Demographics and other covariates

Sexual orientation was assessed from a question that invited respondents to select the category that best identified their sexual identity. Respondents were classified as lesbian identified or bisexual identified (the latter including respondents who identified as bisexual, pansexual, or other non-monosexual, sexual minority identity). Other demographic variables included the following: age (in years), race/ethnicity (Black/African American, Latinx/Hispanic, White, and all others); educational attainment (college graduate vs. less than college graduate), employment status (employed vs. not employed), relationship status (partnered [married, cohabiting] vs. non-partnered [single, divorced, widowed]; see Table 1). We also constructed a three-category variable for age (18–29, 30–49, 50+) to increase interpretability. Because prior research suggests living in a region with a higher density of same-sex couples confers protection against substance use and psychiatric disorders among sexual minorities (Hatzenbuehler et al., 2014, 2015; Titus et al., 2021), covariates also included a state social climate variable of the proportion (0 to 100%) of same-sex households, based on data from the American Community Survey.

### 2.3. Analysis

All analyses were conducted in Stata (version 17). Bivariate analyses were conducted using Chi-square tests, ANOVA, *t*-tests, and correlation analyses. Number of HED days was analyzed using linear regression, and dichotomous outcomes (weekly marijuana use and weekly tobacco use) were analyzed using logistic regression. Standard errors were clustered at the state level in all multivariate models.

We also conducted two sensitivity analyses. First, we stratified the main analysis for lesbian and bisexual respondents. Second, we repeated the analyses using alternate coding for state policies pertaining to sexual minorities, comparing states with one or more of the three negative policies (*n* = 25) to states without such policies (*n* = 25 and the District of Columbia), to examine whether participant outcomes differed based on the presence of state-level policies that permit denial of services based on sexual orientation, allow denial of adoption or foster parenting by same-sex couples, and/or prohibit or rescind local policies to protect the rights of sexual minorities.

## 3. Results

### 3.1. Mean 4+ drinking days

In bivariate (Table 2) and multivariate analyses (Table 3), comprehensive state policy protections for sexual minorities were significantly associated with fewer days of HED among SMW. Lower educational attainment was associated with more HED days in both bivariate and multivariate analyses. Number of drinking days did not differ among participants based on whether they lived in a state with a state-run alcohol distribution system.

### 3.2. Weekly tobacco use

Bivariate analyses revealed a significantly lower proportion of respondents reporting weekly or more frequent tobacco use in states with comprehensive protections compared to those without such strong policies (11.90% vs 18.16%), but these differences did not hold in the multivariate model. Similarly, bivariate analyses also revealed a significantly lower percent of respondents reporting weekly tobacco use in states with comprehensive smoke-free workplace policies (11.90% vs 20.70%), but these differences also did not hold in the multivariate model. In bivariate analyses, lower education, partnered (vs. not partnered) relationship status, and being age 30–49 was associated with weekly tobacco use, however only lower education remained a demographic predictor of greater odds of weekly tobacco use in the multivariate model.

### 3.3. Weekly marijuana use

Marijuana use was associated with state policy allowing recreational marijuana use in both bivariate and multivariate analyses. Specifically, SMW living in states with policies permitting recreational marijuana use had approximately twice the odds of reporting weekly or more marijuana use compared to SMW living in states without such policies. In the bivariate and multivariate analyses, lower education was associated with higher odds of marijuana use, and older age was associated with lower odds of marijuana use.

### 3.4. Sensitivity analyses

In sensitivity analyses stratified by lesbian and bisexual identity, the impact of comprehensive policy protections was more consistently evident among bisexuals (see Supplemental Tables 1 through 4). Living in states with comprehensive policy protections for sexual minorities was associated with fewer HED days for bisexuals. We also found that living in states with larger percentages of same-sex households was associated with more HED days for bisexuals. Having less than a college education was associated with more HED days for both lesbian and bisexual women. Tobacco use was not associated with state policy environments in stratified analyses, but it was associated with lower education among both lesbians and bisexuals, as well as older age among bisexuals. Laws permitting recreational marijuana use (but not comprehensive sexual minority policy protections) were associated with higher odds of weekly marijuana use among bisexual but not lesbian women. Among lesbians, but not bisexuals, lower education was associated with greater odds of weekly marijuana use, and older age with lower odds of weekly marijuana use.

Sensitivity analyses using any negative policies (versus none) as a predictor variable yielded results that were similar to the primary analyses (see Supplemental Tables 5-7). Living in states with one or more negative policies was significantly associated with a greater number of HED days overall and among bisexual women, but not lesbian women. Negative policies were not associated with weekly tobacco or marijuana use. Comprehensive smoke-free laws were significantly associated with lower odds of weekly tobacco use in the full sample and among bisexuals (but not lesbians). Legalization of recreational marijuana use was associated with higher odds of weekly marijuana use in the full sample and among bisexuals (but not lesbians).

## 4. Discussion

The current study examined the impact of state-level policy environment in the U.S. on substance use behaviors among SMW. Specifically, we examined how policy protections for sexual minorities and policies related to regulation of alcohol, tobacco, and marijuana predicted substance use behaviors among SMW. We found that living in a state with comprehensive state policy protections for sexual minorities was associated with fewer HED days among SMW, but state alcohol control policies had no significant impact. In our study, living in a state with comprehensive policy protections for sexual minorities was not significantly associated with weekly tobacco use or weekly marijuana use. State policies legalizing recreational marijuana use were strongly associated with greater odds of weekly (or more frequent) marijuana use and comprehensive smoke-free policies were only marginally protective against tobacco use among SMW.

The protective effect of living in a state with comprehensive policy protections for sexual minorities on number of HED days is important given research has consistently found higher rates of HED among SMW relative to heterosexual women (Hughes et al., 2020). Findings confirm and extend a growing body of literature documenting positive impacts of sexual minority policy protections on physical health (Gonzales and Ehrenfeld, 2018; Solazzo et al., 2018), psychological health (Hatzenbuehler, 2017; Hatzenbuehler et al., 2009; Raifman et al., 2018), and reduced risk for alcohol use disorder (Hatzenbuehler et al., 2010). Our findings were more definitive than those of an earlier study, which drew from a national population probability sample with a limited number of sexual minorities and found only a marginally protective effect of living in a state with comprehensive policy protections on high-intensity drinking (defined as 8 or more drinks at one time for women; Drabble et al., 2021).

Living in a state with comprehensive smoke-free workplace policies was associated with a lower frequency of tobacco use in bivariate analyses, but did not remain significant in the final model (p-value <0.10). The lack of significance in the final model may be related to our sample size, as only a quarter of the sample reported weekly tobacco use. Other studies have suggested that sexual minority adults are more likely to live in areas that have strong smoke-free laws relative to heterosexual adults (Titus et al., 2021). In the current study, a majority of the 258 participants who lived in states with comprehensive policy protections for sexual minorities also lived in states with strong smoke-free workplace policies (*n* = 242), so the number of participants who lived in states with comprehensive policy protections for sexual minorities but without comprehensive smoke-free workplace policies was small (*n* = 16). This likely limited our ability to detect significant differences in tobacco use by state policy context, in contrast to one study that documented a relationship between structural stigma and tobacco use among sexual minorities, even when accounting for tobacco control environment (Titus et al., 2021). It is of interest that comprehensive smoke-free policies were significantly associated with lower tobacco use when we used an indicator of any negative policies impacting sexual minorities instead of comprehensive policy protections. Additional research is needed, including research with large samples of SMW, to better understand associations between different policy contexts and tobacco use.

State policy allowing recreational marijuana use was strongly associated with increased odds of reporting weekly or more marijuana use, but state policy protections for sexual minorities were not significant. Our study found similar patterns to those revealed in a study on state level medical marijuana laws (MML), which found that bisexual and lesbian women in MML states had higher odds of past year marijuana use than SMW counterparts in non-MML states (Philbin et al., 2019). Philbin and colleagues also found a particularly strong association between MML policy and marijuana use among bisexual women. Although differences in weekly marijuana use between bisexual and lesbian women were not significant in primary analyses, stratified analyses suggested that laws permitting recreational marijuana use were associated with greater odds of weekly marijuana use among bisexuals but not lesbians. Further, among lesbians (but not bisexuals), higher education and older age were protective against weekly marijuana use. Although findings should be interpreted with caution given the reduced sample size in stratified analyses of bisexual and lesbian women, our results highlight the need for additional research to better understand how policy contexts and individual factors may differentially impact substance use among subgroups of SMW.

Findings also underscore the importance of considering how state-level policies may amplify existing disparities in marijuana use among SMW relative to heterosexual women. Prior research has suggested perceived availability, as well as subcultural tolerant norms related to marijuana, illicit drug use, and daily heavy drinking, may be important factors in substance use among sexual minorities (Cochran et al., 2012). Interventions focusing on personalized normative feedback and psychoeducation, including education about social and commercial marketing strategies that capitalize on social identity (Boyle et al., 2020), may be beneficial tools for reducing substance use among sexual minorities. Future research might also explore ways that environmental influences, such as state policies or targeted marketing of legal substances, interact with individual factors, such as perceived norms or use of substances to cope, to predict substance use (Boyle et al., 2020). Research has found that support for sexual minority rights (e.g., legalization of marriage for same-sex couples) and public opinion favoring legalization of marijuana often occur in tandem (Schnabel and Sevell, 2017). Future studies might explore whether policy and social climate related to marijuana use differentially impact SMW.

### 4.1. Limitations

Findings should be interpreted in the context of study limitations. Although the sample was drawn from a large panel sample of SMW across the U.S., participants were not recruited using probability sampling methods. Therefore, the findings may have limited generalizability. Further, despite a relatively large sample, the number of participants living in states with comprehensive policy protections for sexual minorities but weak smoke-free policies was small. Numbers were also small for participants who lived in states with both comprehensive policy protections for sexual minorities and alcohol control policies (*n* = 22), as well as for participants who lived in states with both limited or no comprehensive policy protections for sexual minorities and laws allowing recreational marijuana use (*n* = 20).

Additionally, because the focus of this study was assessing the effects of comprehensive policy environments on substance use, we did not assess the potential impact of implementation of specific policies that protect, or undermine protections, by sexual orientation or gender identity. Although we adopted methods to create a policy index which captured both positive and negative policies to construct our comprehensive policy variable, this approach limits examination of the potential effects of these policies (whether they be positive or negative) separately. This should be an important avenue for future studies, as research examining the impact of the implementation of even one negative policy on different health outcomes is important (Raifman et al., 2018).

There were also some limitations related to measurement in the current study. We assessed frequency of cannabis use, but did not assess the quantity or potency of marijuana during usage days. Although we selected state level policy variables with robust relationships with our outcomes of interest, there are other state policies (e.g., medical marijuana laws, taxes on tobacco products, or open-carry alcohol laws) that might be addressed in future research. In addition, the current study focused on state-level policy, but community level social climate, which was not assessed in the current study, also may be an important predictor of health outcomes among sexual minority populations (Hatzenbuehler et al., 2012; Woodford et al., 2015). There may be other unmeasured factors important to understanding substance use in the context of differing policy environments as well. For example, one study found higher levels of state-level structural stigma (i.e., stigmatizing policies and negative public opinion) and sexual minority rejection sensitivity both predicted tobacco use among sexual minority men (Pachankis et al., 2014). Future studies focusing on the impact of policy context on substance use among SMW might also include measures that assess individual level experiences of stigma. Finally, our focus on past year substance use does not capture how everyday experiences of stigma may predict more immediate behavior; other study designs (e.g., using daily dairy methods) may better document how stigmatizing environments and interactions may both impact daily use.

### 4.2. Conclusions

Our study helps address the paucity of research specific to substance use among SMW, controlling for other state policy and climate factors, and affirms the important role of supportive state policies as a factor that may alleviate disparities in excessive alcohol use among SMW relative to heterosexual women. Findings underscore the importance of policy protections for sexual minorities in reducing substance use, particularly HED, among SMW.

## Supplementary Material

1

## Figures and Tables

**Table 1:** Sample Characteristics (*N* = 732).

	%	*n*
Sexual orientation		
Lesbian	59.70	437
Bisexual	40.30	295
Race/Ethnicity		
White	37.43	274
Black	25.82	189
Latinx	31.15	228
Other/Missing	5.60	41
Educational attainment		
Less than college graduate	54.64	400
College graduate	45.36	332
Employment status		
Employed	75.55	553
Not employed	24.45	179
Relationship status		
Partnered	68.99	505
Not partnered	31.01	227
State policy context		
Comprehensive policy protections	35.10	258
Limited or no policy protections	64.90	477
Tobacco use (regular, 12 month)	15.94	113
Marijuana use (regular, 12 month)	21.31	156
Continuous variables	Mean	SD
Number of days drinking 4+	34.67	81.42
Age	35.45	13.45
Percentage of same-sex households in state	0.37	0.10

SD, standard deviation.

**Table 2:** Bivariate analyses of past 12 months substance use by demographics.

	4+ Drinking Days M (SE)	Weekly Tobacco Use^b^% (n)	Weekly Marijuana Use^b^% (n)
Sexual minority state policies			
Comprehensive policy protections	26.26 (4.24)*	11.90 (30)*	23.64 (61)
Limited or no policy protections	39.26 (4.02)	18.16 (83)	20.04 (95)
Substance-specific policies ^a^			
Present	39.31 (7.44)	11.90 (42)**	26.57 (55)*
Not present	33.42 (3.50)	20.70 (59)	19.33 (87)
Sexual orientation			
Lesbian	31.27 (3.59)†	14.15 (60)	21.05 (92)
Bisexual	39.71 (5.23)	18.60 (53)	21.69 (64)
Race/Ethnicity			
White	27.71 (4.35)	15.30 (41)	17.15 (47)
Black	38.13 (6.50)	16.30 (30)	24.34 (46)
Latinx	40.09 (5.60)	16.89 (37)	23.25 (53)
Other/Missing	35.17 (13.21)	13.16 (5)	24.39 (10)
Educational attainment			
Less than college graduate	45.89 (4.80)***	22.54 (87)***	25.75 (103)**
College graduate	21.16 (3.11)	8.05 (26)	15.96 (53)
Employment status			
Employed	34.48 (3.34)	16.01 (86)	21.16 (117)
Not employed	35.27 (6.72)	15.70 (27)	21.79 (39)
Relationship status			
Partnered	36.23 (3.70)	17.85 (88)*	21.19 (107)
Not partnered	31.22 (5.14)	11.57 (25)	21.59 (49)
Age			
18–29	33.87 (78.81)	13.94 (40)* ^2^	24.50 (74)** ^3^
30–49	42.24 (89.38)	20.00 (59)	22.19 (67)* ^3^
50+	17.63 (63.44)* ^1^	10.48 (13)* ^2^	12.00 (15)
Continuous variables	Correlation	M (SD)	M (SD)
Age	−0.05	35.51 (11.77)	32.71 (10.28)
Percent of same-sex households in state	.02	0.36 (0.06)	0.37 (0.09)

*** *p*<.001

** *p*< .01

* *p* <0.05

† *p*<.01.

M, mean. SE, standard error.

1 50+ group significantly different than 30–39 group.

2 30–49 age group significantly greater than 18–29 group and 50+ group.

3 50+ group significantly lower than 30–49 and 18–29 age groups.

a Substance-specific policies included the following: state wholesale or retail alcohol control policies (used in analysis of 4+ drinking days); comprehensive smoke-free policies (used in analysis of weekly tobacco use); recreational marijuana use allowed by policy (used in analysis of weekly marijuana use).

b Weekly use or more often.

**Table 3:** Multivariate associations of past 12 months substance use with state policy environment and proportion of same-sex households.

	4+ Drinking Days b (SE)	Weekly Tobacco Use ^a^OR (95% CI)	Weekly Marijuana Use ^a^OR (95% CI)
State policy protections for sexual minorities	**−15.11 (7.27)**	1.12 (0.52 - 2.42)	0.96 (0.50–1.85)
	***p*=.038**	*p*=.768	*p*=.898
Substance-specific policies^1^	8.45 (8.12)	0.54 (0.28–1.04)†	**2.14 (1.09–4.23)**
	*p*=.298	*p*=.064	***p*=.028**
Percentage of same-sex householders in state	65.98 (34.69)†	0.610 (0.03- 12.34)	0.585 (0.06, 5.64)
	*p*=.057	*p*=.747	*p*=.643
Education^2^	**24.24 (6.83)**	**3.71 (2.18 - 6.31)**	**1.70(1.11–2.61)**
	***p*=<0.001**	***p*= <0.001**	***p*=.015**
Unpartnered	−2.00 (7.81)	0.63 (0.37–1.07)	1.10 (0.72–1.67)
	*p*=.770	*p*=.09†	*p*=.657
Age	0.06 (0.25)	1.01 (0.99 - 1.03)	**0.975 (0.96–0.99)**
	*p*=.804	*p*=.168	***p*=.004**

Notes:.

Multivariate models also adjusted for the following variables that were not significant in any analyses: sexual orientation, race/ethnicity, employment, and relationship status.

1 Substance specific policies included the following: state wholesale or retail alcohol control policies (used in analysis of 4+ drinking days, ref group=no state alcohol control policies); comprehensive smoke-free policies (used in analysis of weekly tobacco use, ref group=states without comprehensive smoke-free policies); recreational marijuana use allowed by policy (used in analysis of weekly marijuana use, ref group=states without recreational marijuana use).

2 Less than college graduate (ref group: college graduates).

a Weekly use or more often.
