# Corrigendum to ‘Impact of the policy environment on substance use among sexual minority women’

**DOI:** 10.1016/j.dadr.2022.100080

**Published:** 2022-07-11

**Authors:** Laurie A. Drabble, Cat Munroe, Amy A. Mericle, Sarah Zollweg, Karen F. Trocki, Katherine J. Karriker-Jaffe

**Affiliations:** a San Jose State University College of Health and Human Sciences, San Jose, CA, USA; b Alcohol Research Group, Public Health Institute, Emeryville, CA, USA; c Columbia University School of Nursing; d Community Health & Implementation Research Program, RTI International, Berkeley, CA, USA

The authors regret the Author Disclosure information was missing from the above-mentioned published article. The information is now reproduced below. The authors would like to apologise for any inconvenience caused.

## Declaration of Competing Interest

No conflict declared.

