# Supplementary material for: Impact of the policy environment on substance use among sexual minority women
Source: Drug Alcohol Depend Rep. 2022 Apr 29;3:100058. doi: 10.1016/j.dadr.2022.100058 (PMC9262325; doi:10.1016/j.dadr.2022.100058)
Supplement: Supplementary file 1 [file mmc1.docx]

**Supplemental Table 1**

*Sample Characteristics by Sexual Orientation (N=732)*

| Variable | Lesbian | | Bisexual | |
| --- | --- | --- | --- | --- |
|  | *n* | % | *n* | % |
| Age | | | | |
| 18-29 | 158 | 36.16 | 144 | 49.32 |
| 30-49 | 178 | 40.73 | 124 | 42.47 |
| 50+ | 101 | 23.11 | 24 | 8.22 |
| Race/Ethnicity | | | | |
| White | 152 | 34.78 | 122 | 41.36 |
| Black | 124 | 28.38 | 65 | 22.03 |
| Latinx | 138 | 31.58 | 90 | 30.51 |
| Other/Missing | 23 | 5.26 | 18 | 6.10 |
| Educational Attainment | | | | |
| Some college or less | 204 | 46.68 | 196 | 66.44 |
| College graduate | 233 | 53.32 | 99 | 33.56 |
| Employment status | | | | |
| Employed | 350 | 80.09 | 203 | 68.81 |
| Not employed | 87 | 19.91 | 92 | 31.19 |
| Relationship status | | | | |
| Partnered | 300 | 68.65 | 205 | 69.49 |
| Not partnered | 137 | 31.35 | 90 | 30.51 |
| Tobacco use | | | | |
| Once per week or more | 60 | 14.15 | 53 | 18.60 |
| Less often than weekly | 364 | 85.85 | 232 | 81.40 |
| Marijuana use | | | | |
| Once per week or more | 92 | 21.05 | 64 | 21.69 |
| Less often than weekly | 345 | 78.95 | 231 | 78.31 |
| State policy context | | | | |
| All 5 positive policies and no negative policies | 153 | 35.01 | 105 | 35.59 |
| Less than 5 positive policies | 284 | 64.99 | 190 | 64.41 |

Title: Impact of the Policy Environment on Substance Use among Sexual Minority Women

**Supplemental Table 2**

*Linear regression models for number of 4+ drinking days in past 12 months, by positive state sexual minority policy environment and demographics*

| Variable | Total sample (*N*=732) | | Lesbian  (*n*=437) | | Bisexual  (*n*=295) | |
| --- | --- | --- | --- | --- | --- | --- |
|  | *b* | *(SE)* | *b* | *(SE)* | *b* | *(SE)* |
| Comprehensive policy protections | -15.114* | 7.266 | -1.247 | 8.734 | -37.168** | 12.895 |
| Alcohol control state | 8.447 | 8.119 | 10.739 | 9.824 | 3.891 | 13.941 |
| Proportion same sex households * 100 | 65.984† | 34.686 | 30.080 | 37.090 | 176.499* | 78.185 |
| Bisexual | 4.269 | 6.672 |  |  |  |  |
| Age | 0.063 | 0.252 | -0.196 | 0.291 | 0.419 | 0.492 |
| Black | 9.736 | 7.900 | 4.303 | 9.493 | 19.449 | 13.898 |
| Latinx | 12.166 | 7.731 | 13.040 | 9.534 | 10.993 | 12.845 |
| Some college or less | 24.244*** | 6.830 | 25.421** | 8.209 | 23.152* | 11.819 |
| Unemployed | -5.323 | 7.727 | -9.610 | 9.896 | 1.311 | 12.191 |
| Not partnered | -1.996 | 6.813 | 7.173 | 8.171 | -16.625 | 11.907 |

* *p*≤.05, ** *p*<.01, *** *p*<.001 † *p*<.10

Reference: Lesbian, white/other, college graduate, employed, partnered.

**Supplemental Table 3**

*Logistic regression models for weekly tobacco use^a^ in past 12 months, by positive state sexual minority policy environment and demographics*

| Variable | Total sample (*N*=732) | | Lesbian  (*n*=437) | | Bisexual  (*n*=295) | |
| --- | --- | --- | --- | --- | --- | --- |
|  | OR | 95% CI | OR | 95% CI | OR | 95% CI |
| Comprehensive policy protections | 1.122 | (0.521, 2.417) | 1.631 | (0.544, 4.894) | 0.849 | (0.286, 2.522) |
| Smoke-free policies | 0.543† | (0.285, 1.036) | 0.486 | (0.190, 1.242) | 0.648 | (0.262, 1.597) |
| Proportion same sex households * 100 | 0.610 | (0.0301, 12.34) | 0.066 | (0.000427, 10.06) | 6.296 | (0.0851, 466.0) |
| Bisexual | 1.374 | (0.866, 2.179) |  |  |  |  |
| Age | 1.013 | (0.995, 1.032) | 0.997 | (0.972, 1.024) | 1.037* | (1.008, 1.067) |
| Black | 0.880 | (0.500, 1.548) | 0.659 | (0.288, 1.508) | 1.055 | (0.466, 2.391) |
| Latinx | 0.923 | (0.530, 1.609) | 1.189 | (0.544, 2.600) | 0.615 | (0.273, 1.384) |
| Some college or less | 3.710*** | (2.181, 6.312) | 4.674*** | (2.255, 9.688) | 2.824* | (1.273, 6.267) |
| Unemployed | 0.685 | (0.394, 1.191) | 0.561 | (0.235, 1.342) | 0.815 | (0.387, 1.716) |
| Not partnered | 0.633† | (0.374, 1.071) | 0.686 | (0.328, 1.432) | 0.639 | (0.293, 1.393) |

* *p*<.05, ** *p*<.01, *** *p*<.001, † *p*<.10

Reference: Lesbian, white/other, college graduate, employed, partnered.

*^a^ Use of tobacco once a week or more often (dichotomous indicator variable).*

**Supplemental Table 4**

*Logistic regression models for weekly marijuana use^a^ in past 12 months, by positive state sexual minority policy environment and demographics*

| Variable | Total sample (*N*=732) | | Lesbian  (*n*=437) | | Bisexual  (*n*=295) | |
| --- | --- | --- | --- | --- | --- | --- |
|  | OR | *95% CI* | OR | *95% CI* | OR | *95% CI* |
| Comprehensive policy protections | 0.958 | (0.496, 1.849) | 1.177 | (0.522, 2.656) | 0.627 | (0.231, 1.701) |
| Recreational marijuana use allowed by policy | 2.143** | (1.086, 4.229) | 1.674 | (0.705, 3.975) | 2.988* | (1.144, 7.804) |
| Proportion same sex households * 100 | 0.585 | (0.061, 5.637) | 0.361 | (0.020, 6.392) | 2.111 | (0.036, 123.7) |
| Bisexual | 0.946 | (0.631, 1.417) |  |  |  |  |
| Age | 0.975*** | (0.958, 0.992) | 0.971*** | (0.951, 0.993) | 0.984 | (0.955, 1.014) |
| Black | 1.304 | (0.797, 2.136) | 1.152 | (0.591, 2.247) | 1.414 | (0.665, 3.011) |
| Latinx | 1.033 | (0.643, 1.661) | 1.231 | (0.652, 2.323) | 0.792 | (0.379, 1.655) |
| Some college or less | 1.699** | (1.107, 2.608) | 2.362*** | (1.354, 4.118) | 1.044 | (0.536, 2.032) |
| Unemployed | 0.856 | (0.531, 1.381) | 1.112 | (0.582, 2.125) | 0.658 | (0.322, 1.342) |
| Not partnered | 1.100 | (0.722, 1.674) | 1.134 | (0.652, 1.973) | 1.067 | (0.550, 2.068) |

* *p*<.05, ** *p*<.01, *** *p*<.001, † *p*<.10*^a^ Use of marijuana once a week or more often (dichotomous indicator variable).*

Reference: Lesbian, white/other, college graduate, employed, partnered.

**Supplemental Table 5**

*Linear regression models for number of 4+ drinking days in past 12 months, by any negative state sexual minority policy and demographics*

| Variable | Full sample  (*N*=732) | | Lesbian  (*n*=437) | | Bisexual  (*n*=295) | |
| --- | --- | --- | --- | --- | --- | --- |
|  | *b* | *(se)* | *b* | *(se)* | *b* | *(se)* |
| Any negative state policy | 14.37* | (6.729) | 2.859 | (8.144) | 29.648* | (11.956) |
| Alcohol control state | 9.439 | (8.061) | 10.494 | (9.777) | 8.792 | (13.970) |
| Proportion same sex households * 100 | 61.40† | (33.889) | 31.598 | (36.303) | 148.711† | (76.136) |
| Bisexual | 4.095 | (6.666) |  |  |  |  |
| Age | 0.056 | (.252) | -0.193 | (0.292) | 0.373 | (0.497) |
| Black | 10.249 | (7.869) | 4.221 | (9.449) | 20.727 | (13.926) |
| Latinx | 11.291 | (7.726) | 12.942 | (9.538) | 7.452 | (12.826) |
| Some college or less | 24.420*** | (6.820) | 25.295** | (8.188) | 23.576* | (11.865) |
| Unemployed | -4.761 | (7.744) | -9.386 | (9.918) | 1.738 | (12.254) |
| Not partnered | -2.330 | (6.810) | 7.018 | (8.180) | -15.553 | (11.992) |
| * *p*<.05, ** *p*<.01, *** *p*<.001, † *p*<.10 | |  |  |  |  |  |
|  | |  |  |  |  |  |

Reference: Lesbian, white/other, college graduate, employed, partnered.

**Supplemental Table 6**

*Logistic regression models for weekly tobacco use^a^ in past 12 months, by any negative state sexual minority policy and demographics*

| Variable | Full sample  (*N=732)* | | Lesbian  (*n*=437) | | Bisexual  (*n*=295) | |
| --- | --- | --- | --- | --- | --- | --- |
|  | OR | 95% CI | OR | 95% CI | OR | 95% CI |
| Any negative state policy | 0.698 | (0.365, 1.335) | 0.597 | (0.217, 1.645) | 0.585 | (0.231, 1.482) |
| Smoke-free policies | 0.467* | (0.250, 0.874) | 0.458 | (0.176, 1.192) | 0.432* | (0.182, 1.028) |
| Proportion same sex households * 100 | 0.415 | (0.0221, 7.797) | 0.074 | (0.001, 8.898) | 1.854 | (0.030, 113.952) |
| Bisexual | 1.373 | (0.866, 2.177) |  |  |  |  |
| Age | 1.013 | (0.995, 1.032) | 0.997 | (0.971, 1.023) | 1.039** | (1.010, 1.069) |
| Black | 0.880 | (0.500, 1.547) | 0.636 | (0.277, 1.463) | 1.123 | (0.495, 2.549) |
| Latinx | 0.940 | (0.540, 1.636) | 1.207 | (0.552, 2.639) | 0.641 | (0.283, 1.452) |
| Some college or less | 3.729*** | (2.195, 6.332) | 4.690*** | (2.265, 9.710) | 2.829** | (1.275, 6.277) |
| Unemployed | 0.672 | (0.387, 1.169) | 0.547 | (0.228, 1.311) | 0.786 | (0.372, 1.663) |
| Not partnered | 0.635† | (0.375, 1.074) | 0.707 | (0.337, 1.481) | 0.609 | (0.277, 1.341) |
| *Note*. CI = confidence interval. | |  |  |  |  |  |
| * *p*<.05, ** *p*<.01, *** *p*<.001, † *p*<.10 | |  |  |  |  |  |

Reference: Lesbian, white/other, college graduate, employed, partnered.

*^a^ Use of tobacco once a week or more often (dichotomous indicator variable).*

**Supplemental Table 7**

*Logistic regression models for weekly marijuana use^a^ in past 12 months, by any negative state sexual minority policy and demographics*

| Variable | Full sample  (*N*=732) | | Lesbian  (*n*=637) | | Bisexual  (*n*=295) | |  |
| --- | --- | --- | --- | --- | --- | --- | --- |
|  | OR | 95% CI | OR | 95% CI | OR | 95% CI |  |
| Any negative state policy | 1.415 | (0.844 - 2.373) | 1.128 | (0.573 - 2.222) | 2.093† | (0.919 - 4.769) |  |
| Recreational marijuana use allowed by state policy | 2.372** | (1.307 - 4.304) | 1.981† | (0.919 - 4.270) | 3.226** | (1.395 - 7.458) |  |
| Proportion same sex households * 100 | 0.758 | (0.0894 - 6.423) | 0.446 | (0.0277 - 7.173) | 2.81 | (0.0505 - 156.0) |  |
| Bisexual | 0.953 | (0.637 - 1.427) |  |  |  |  |  |
| Age | 0.975** | (0.958 - 0.991) | 0.972** | (0.951 - 0.993) | 0.98 | (0.952 - 1.011) |  |
| Black | 1.28 | (0.784 - 2.090) | 1.146 | (0.589 - 2.232) | 1.37 | (0.641 - 2.914) |  |
| Latinx | 1.014 | (0.631 - 1.629) | 1.222 | (0.647 - 2.309) | 0.76 | (0.367 - 1.588) |  |
| Some college or less | 1.679* | (1.096 - 2.572) | 2.332** | (1.338 - 4.065) | 1.03 | (0.528 - 2.009) |  |
| Unemployed | 0.878 | (0.544 - 1.415) | 1.142 | (0.596 - 2.187) | 0.67 | (0.325 - 1.368) |  |
| Not partnered | 1.098 | (0.722 - 1.670) | 1.132 | (0.651 - 1.969) | 1.12 | (0.575 - 2.180) |  |
| *Note*. CI = confidence interval. | | | |  |  |  |  |
| * *p*<.05, ** *p*<.01, *** *p*<.001, † *p*<.10 | | | |  |  |  |  |

Reference: Lesbian, white/other, college graduate, employed, partnered.

*^a^ Use of marijuana once a week or more often (dichotomous indicator variable).*
